# Unveiling Microplastics in Commercial Brackish Water Fishes from the Lower Meghna River Estuary of Bangladesh

**DOI:** 10.1007/s00128-025-04048-3

**Published:** 2025-04-26

**Authors:** Razat Suvra Das, S. M. Mahatab Uddin, Sedat Gündoğdu, Jannatul Kubra Afrin, Nusrat Jahan, Md. Rubaet Bin Abedin, Showmitra Chowdhury, Sultan Al Nahian, M. Golam Mustafa, Mohammad Abdul Momin Siddique

**Affiliations:** 1 Department of Oceanography, Noakhali Science and Technology University, Noakhali, 3814 Bangladesh; 2 Department of Basic Sciences, Faculty of Fisheries, Cukurova University, Adana, 01330 Turkey; 3 Bangladesh Oceanographic Research Institute, Cox’s Bazar, 4730 Bangladesh; 4 Faculty of Fisheries and Protection of Waters, South Bohemian Research Center of Aquaculture and Biodiversity of Hydrocenoses, Research Institute of Fish Culture and Hydrobiology, University of South Bohemia in Ceske Budejovice, Zatisi 728/II, Vodnany, 389 25 Czech Republic

**Keywords:** Microplastic pollution, Brackish water fishes, Aquatic ecosystem, Meghna estuary

## Abstract

Microplastics (MPs) pose a persistent global threat and have entered almost every component of the ecosystem and seafood items. This study aimed to identify and characterize MPs in three widely edible fishes in the lower Meghna River estuary of Bangladesh and assess the polymer hazard index. Gastrointestinal tracts (GIT) of fish were collected and digested with KOH to extract MPs, and the polymer was confirmed by FTIR analysis. The species with the highest mean MP abundance was *M. gulio* (22.89 ± 8.91 MPs/GIT), followed by *P. paradiseus* (10.78 ± 4.29) and *O. pama* (15.11 ± 3.55). Fibers were predominant MPs, comprising 73.20–91.75% of the total particles. Blue, red, and black were the dominant colors of MPs, while 81.07–93.81% particles were between 500 and 1000 μm. Five distinct polymers, polypropylene, polyethylene, polyethylene terephthalate, Polystyrene, and Nylon 6, were detected in the GIT of these fishes. The study unveiled a significant correlation (r^2^ = 0.223, *p* = 0.013) between MP incidence and the total length of fish. The polymer hazard index of the selected fishes showed the risk category IV (Danger), which is alarming. A risk category IV indicates that exposure to polymer, directly or indirectly through the food chain, may result in severe health consequences for humans as well as wildlife. With these detailed insights into MPs in most consumable fishes, the study highlights the comprehensive risks posed by MPs that could be useful for strategies to mitigate this environmental challenge.

**Supplementary Information:**

The online version contains supplementary material available at 10.1007/s00128-025-04048-3.

## Introduction

Plastic has replaced traditional materials such as glass, wood, and pottery due to its convenience, durability, and affordability. Despite being widely recognized as a pollutant, plastic poses a serious environmental hazard to aquatic organisms and ecosystems (Walker 2018). Marine plastic pollution is a significant issue due to its widespread presence and long-lasting environmental impact. It affects marine species, ecosystems, the tourism sector, fisheries, maritime infrastructure, and the safety of mariners at sea (Karbalaei et al. 2018). MPs have been reported for various ecosystems, such as freshwater bodies, marine environments, deep-sea habitats, coastal sediments, and beach areas (Rahman et al. 2020; Pinheiro et al. 2023).

MPs can travel long distances due to their small size and low density (Barboza et al. 2019). Fish is one of the major foods that we consume around the world. In their natural habitat, fish can ingest MPs unintentionally through water intake by mouth and gills, consuming MP-contaminated plankton and small fishes, and intentionally as food (Li et al. 2021; Siddique et al. 2024a). Microplastics have been discovered in fish across various habitats, from freshwater to marine and estuarine environments (Foo et al. 2022; Gad and Midway 2022; Bilal et al. 2023; Gholizadeh et al. 2024; Siddique et al. 2024b).

Estuaries, critical aquatic ecosystems, offer habitat, food, and coastal protection for numerous species, including fish, seabirds, and mammals. These environments are particularly susceptible to pollution and can facilitate the accumulation and spread of MPs. Various sources, such as runoff, municipal waste treatment plants, and sewage discharges, introduce plastic items from rivers into estuaries. Numerous studies have highlighted the significance of estuaries in plastic pollution, showing higher concentrations of MPs in estuarine systems (Amponsah et al. 2024), thus identifying estuaries as primary conduits for MPs entering oceans. Between 1.5 and 2.4 million metric tons of macro- and microplastics are estimated to reach the oceans annually via estuaries worldwide. The annual input of 8 million metric tons of plastic trash into oceans is projected to double by 2030 and quadruple by 2050 (Jambeck et al. 2015).

The lower Meghna River estuary in Bangladesh is a unique geographic region, part of the Ganges-Brahmaputra Delta, with diverse ecosystems and significant ecological value. This estuary provides habitats for various aquatic animals, including fish, crustaceans, and other marine life. The Meghna River estuary faces environmental challenges, such as pollution, overfishing, coastal erosion, and climate change due to human activities (Siddique et al. 2021). Despite its importance, it remains underexplored in terms of microplastic research. Investigating microplastics in commercially important brackish water fish species is a novel approach. While many studies have focused on marine or freshwater fish, brackish waters may present distinct pollution dynamics. This study targets commercially important brackish water fish species Pama croaker *Otolithoides pama*, Long Whiskers Catfish *Mystus gulio*, and Paradise threadfin *Polynemus paradiseus*, which could experience different levels of microplastic contamination compared to marine or freshwater fish. By focusing on these species, the research highlights a direct human health risk, as microplastics can enter the food chain through fish consumption, impacting both local populations and international markets that depend on these species. This approach may provide insights into potential food safety risks, which are becoming an increasingly important concern for global food security.

Previous studies have reported MP contamination in king mackerel *Scomberomorus guttatus* (Hossain et al. 2023) and giant river catfish *Sperata seenghala* (Arafat et al. 2023) from the Meghna River estuary. However, further research is needed to better understand the level of MP contamination in the most consumable and economically valuable fish species from this estuary. Hence, this study aimed to investigate the prevalence of MPs in the gastrointestinal tract of three commonly consumed brackish-water fish species *O. pama*, *M. gulio*, and *P. paradiseus*, from the lower Meghna River estuary.

## Materials and Methods

### Study Area and Sample Collection

**Fig. 1:**
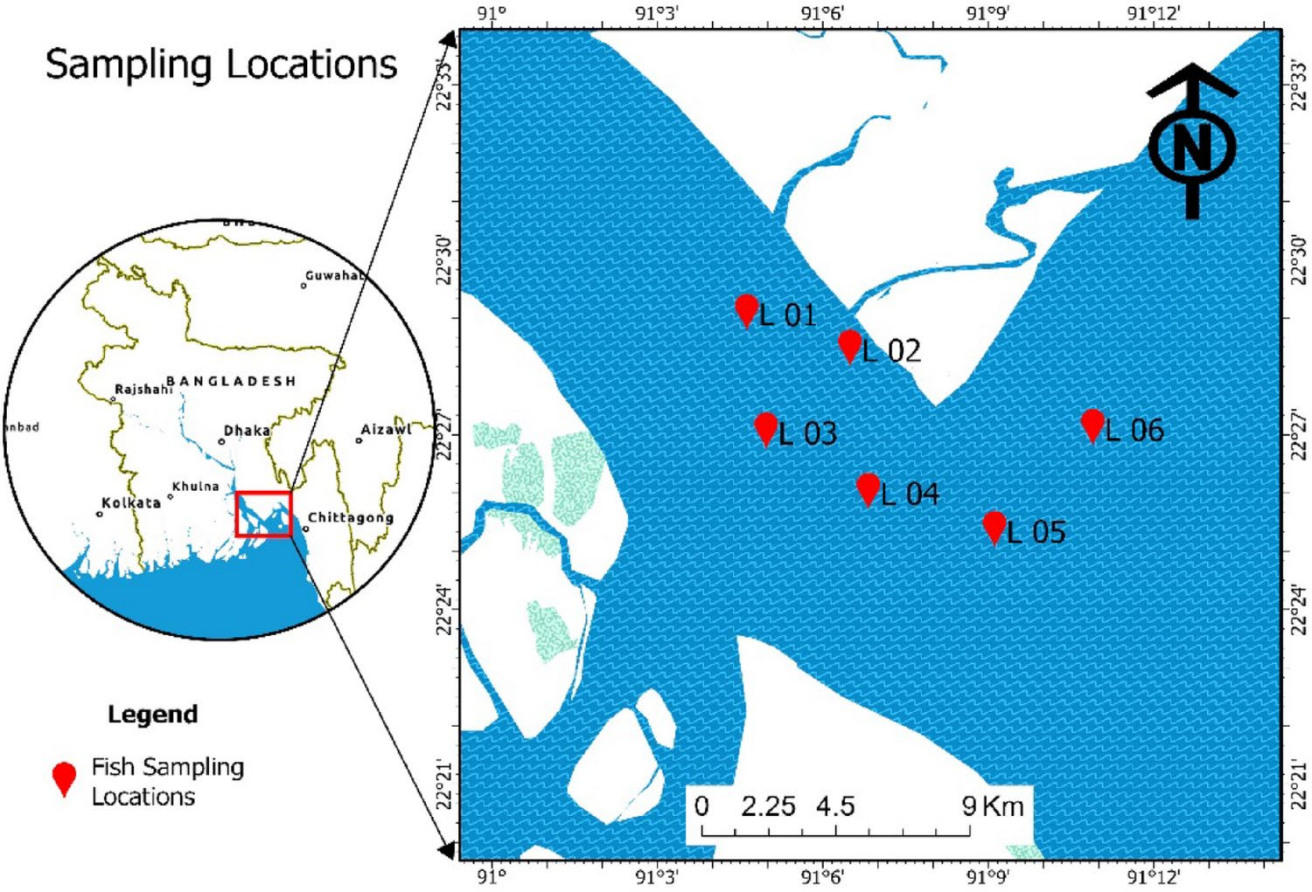
Study area map of the lower Meghna River estuary, Bay of Bengal

Three fish species *O. pama*, *M. gulio*, and *P. paradiseus*, comprising 10 mature individuals per species (30 specimens), were collected from the lower Meghna estuary. Species were chosen based on their high economic value, taste, availability, and preference by the local consumers. Fish samples were collected from the anglers at the sampling sites. Species name, trophic level, habitat, feeding habit, total length, body weight, and gastrointestinal tract (GIT) weight of studied fish species are presented in Table 1. Sampling sites are shown in Fig. 1. Samples were promptly placed in an icebox, transported to the Department of Oceanography, Noakhali Science and Technology University, and stored at − 20 °C for subsequent research use (Siddique et al. 2022).

**Table 1:** Species name, trophic level, habitat, feeding habit, total length, body weight, and Gastrointestinal tract (GIT) weight of studied fish species. Data is presented as mean ± standard deviation

Species name	Trophic level	Habitat	Feeding habit	Total length (cm)	Body weight (g)	GIT weight (g)
*Otolithoides pama*	3.9 ± 0.4	Benthopelagic; Amphidromous	Carnivore	21.17 ± 2.28	72.12 ± 20.81	0.15 ± 0.04
*Mystus gulio*	4.0 ± 0.5	Demersal; Anadromous	Carnivore	12.82 ± 2.28	21.63 ± 10.92	0.49 ± 0.61
*Polynemus paradiseus*	3.9 ± 0.6	Demersal; Amphidromous	Carnivore	16.73 ± 2.62	33.54 ± 18.40	0.32 ± 0.34

### Sample Preparation

All specimens were washed with filtered water to avoid additional MP contamination, and their total lengths and body weight were recorded. Each specimen was dissected individually using scissors, a scalpel, and forceps to extract the gastrointestinal tract (GIT), which was then placed in a 250 mL glass beaker (Siddique et al. 2022). The GIT’s weight was measured after being placed in the glass beaker. GIT tissue was digested with a 10% KOH solution (Feng et al. 2019). KOH was chosen for digestion because it effectively breaks down organic material without altering the natural colors of MPs (Karami et al. 2017). The beakers were then sealed with foil paper and incubated in a controlled oven (Velp Scientifica, Italy) at 40 °C for 24–48 h, depending on the extent of organic material digestion (Avio et al. 2015). Density separation was performed using 4.4 M, 1.5 g/mL of sodium iodide (NaI) solution (Merck, Germany) according to the method followed by Siddique et al. (2025). After density separation, the retained material was washed with filtered-distilled water into a clean glass beaker and then vacuum-filtered using glass microfiber filter membranes (Sartorius; pore size of 0.45 μm) (Siddique et al. 2025). The filters were placed in clean glass petri dishes and dried at room temperature at 30 °C.

### Visual Identification and Characterization

After extraction, filters were inspected using a Labomed CXL-110,446,002, 9,135,002 light binocular microscope (USA) at 16 × 4 and 16 × 10 magnification (Siddique et al. 2022). For contamination prevention, each utensil was thoroughly washed with 70% ethanol and Milli-Q water prior to use. All procedures were performed on a clean bench, and for every batch of assays, a control/blank sample was included (Rahman et al., 2020). Images of MP particles were captured, and each item was characterized by length, shape, and color. According to the physical attributes and availability, MPs were categorized as fragments, fibers, and films. Size of the MPs was categorized into small (< 500 μm), medium (500–1000 μm), and large (1000–5000 μm) (Hossain et al. 2019). Detected MPs displayed a range of colors, including red, green, blue, crystal, brown, and black.

### Fourier-Transform Infrared (FT-IR) Spectroscopy Analysis

Polymers were identified using ATR- FTIR (SHIMADZU IR Spirit A224159). Background scans were conducted to avoid data distortion. MP particles were randomly picked and analyzed using an FT-IR with IR Solution software. The MP particles were identified using a detector’s spectral range of 4000–400 cm^− 1^. Spectra results were assessed and compared using two reference polymer libraries (Shimadzu Thermal Damaged Plastics Library and Shimadzu UV Damaged Plastics Library). FTIR analyses of randomly selected subsamples from all types confirmed 98 synthetic polymer nature. Particles were considered as MPs when the spectrum quality index matched 75% with the library (Cui et al. 2022; Siddique et al. 2023).

### Quality Control and Quality Assurance

The study implemented stringent measures to prevent cross-contamination, covering all processes from sample collection to laboratory analysis. Laboratory access was restricted to avoid MP contamination. Clean cotton lab coats and nitrile gloves were worn, and the workstation and dissection tools were washed with deionized water. Glassware was used for all containers and tools, and aluminum foil and glassware were employed to shield equipment, samples, and filters from airborne particles. All procedures were executed promptly to minimize contamination risks.

### Data Analysis

Data analysis was performed using IBM SPSS Statistics Version 26 and PAST V4.13. MP abundance was reported as MPs/GIT and MPs/g GIT. Before performing the parametric test, the normality of data was verified using Sapiro-Wilk’s test. A one-way ANOVA followed by Tukey’s post-hoc test was conducted to compare the abundance of MPs among species. All statistical tests were performed at an alpha level of 0.05. Regression analysis was conducted to explore correlations between MP quantity and fish length, weight, and GIT weight.

The polymer hazard index (PHI) for consuming MPs-containing fish was determined to assess potential risk for humans. According to the methodology used by Nithin et al. (2022), the PHI value was calculated by the following formula:

1 $$ {\text{PHI}} = \sum {{\text{P}}_{{\text{n}}} \times {\text{S}}_{{\text{n}}} } $$

Where P_n_ is the percentage of specific plastic polymers, and S_n_ is the hazard score of plastic polymers obtained from Lithner et al. (2011).

## Result

### Abundance of Microplastics

**Fig. 2:**
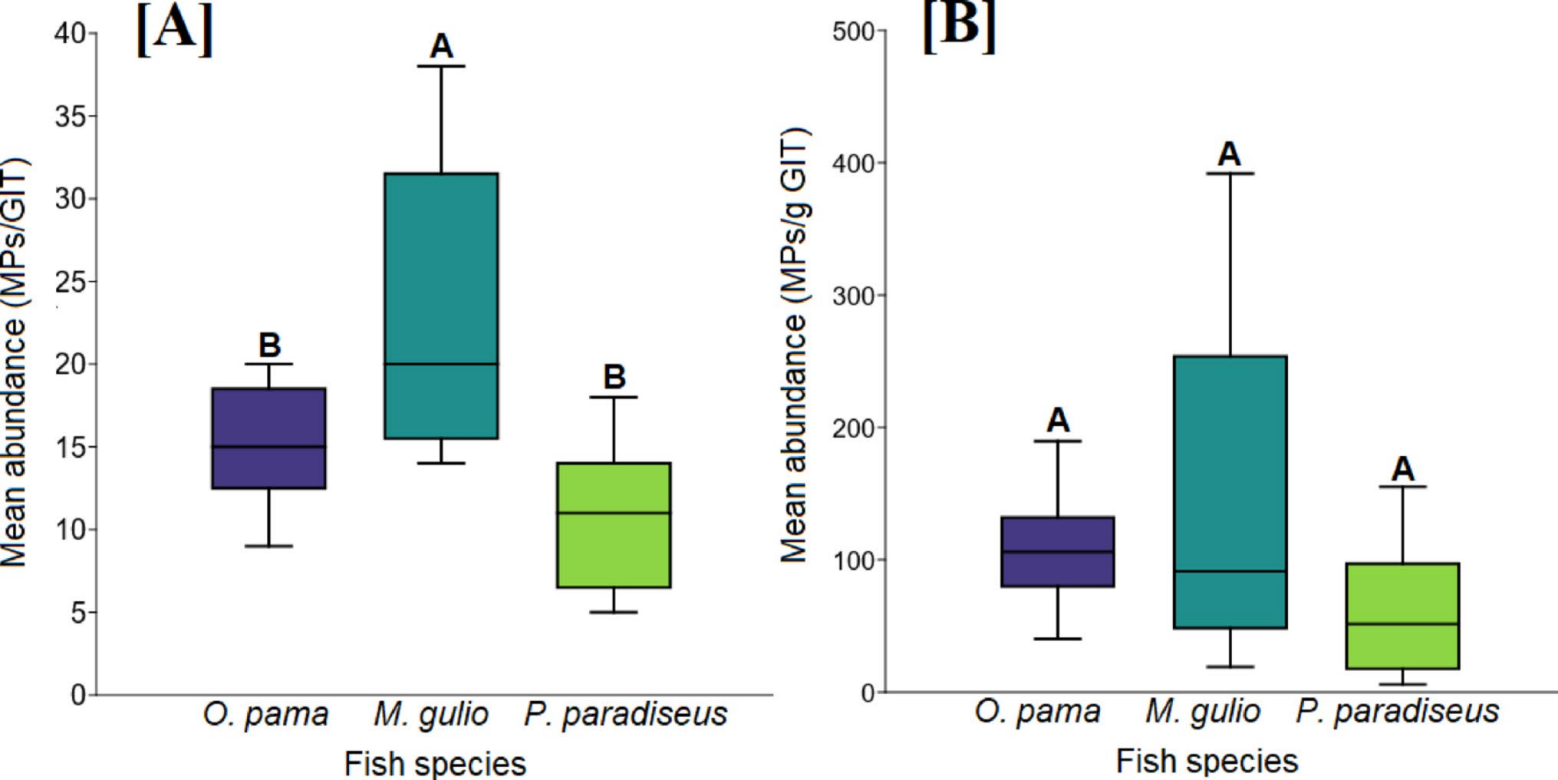
Microplastic abundance in three estuarine fish species from the Meghna River estuary. **A** shows the mean abundance of microplastics per fish GIT, and **B** shows the mean abundance of microplastics per gram of fish GIT. Data are presented as mean values with standard deviation. Different letters on the bars indicate significant differences in MP abundance among species (*p* < 0.05)

The presence of MPs was 100% in the three examined fish species collected from the Meghna River estuary. A total of 439 MP particles were extracted from *O. pama*,* M. gulio*, and *P. paradiseus samples.* The highest abundance was observed in *M. gulio*, with 206 MPs (22.89 ± 8.91 MPs/GIT), followed by *O. pama* with 136 MPs (15.11 ± 3.55 MPs/GIT), and *P. paradiseus*, which had 97 MPs in their GITs (10.78 ± 4.29 MPs/GIT) (Fig. 2A). One-way ANOVA showed a significant variation in MP abundance (F = 9.209, *p* = 0.001) among the species when we computed the MP abundance per GIT (Fig. 2A). However, when we estimated the abundance per gram of GIT, no significant differences were observed among the species (F = 2.104, *p* = 0.144) (Fig. 2B).

### Characteristics of Microplastics

**Fig. 3:**
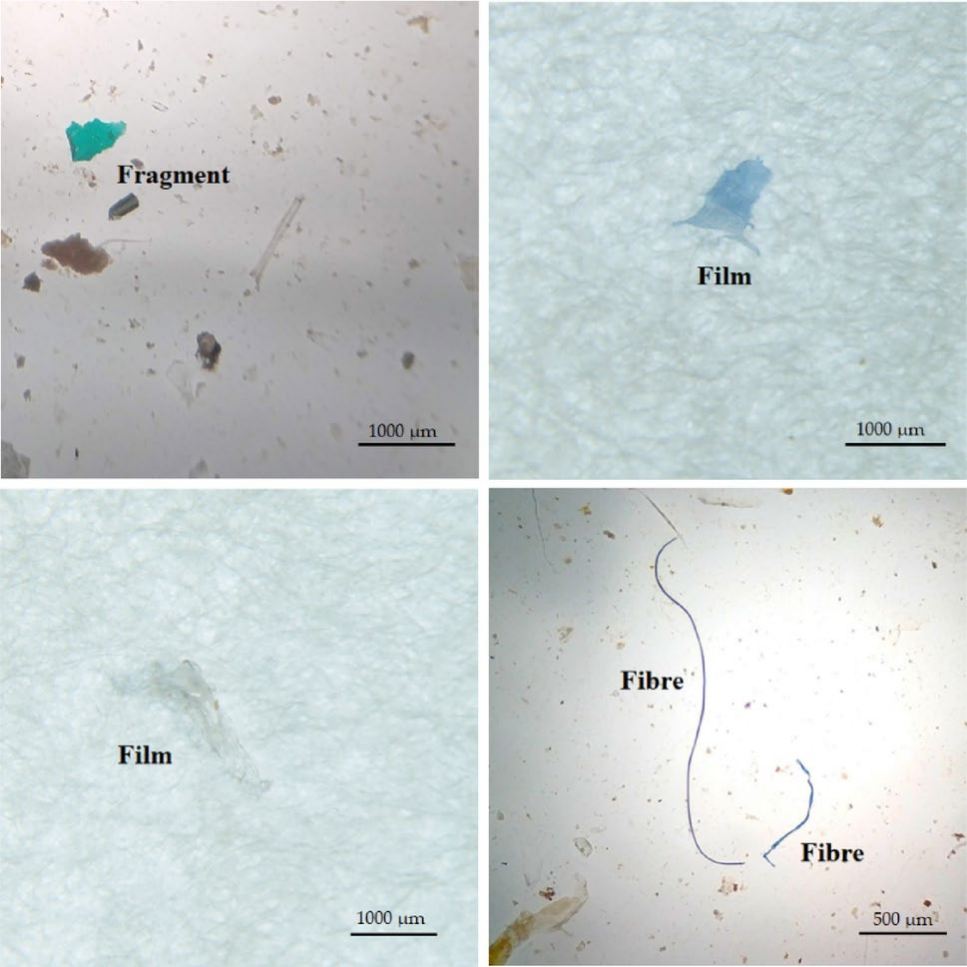
Selected photographs of identified MPs in *O. pama*,* M. gulio*, and *P. paradiseus*

**Fig. 4:**
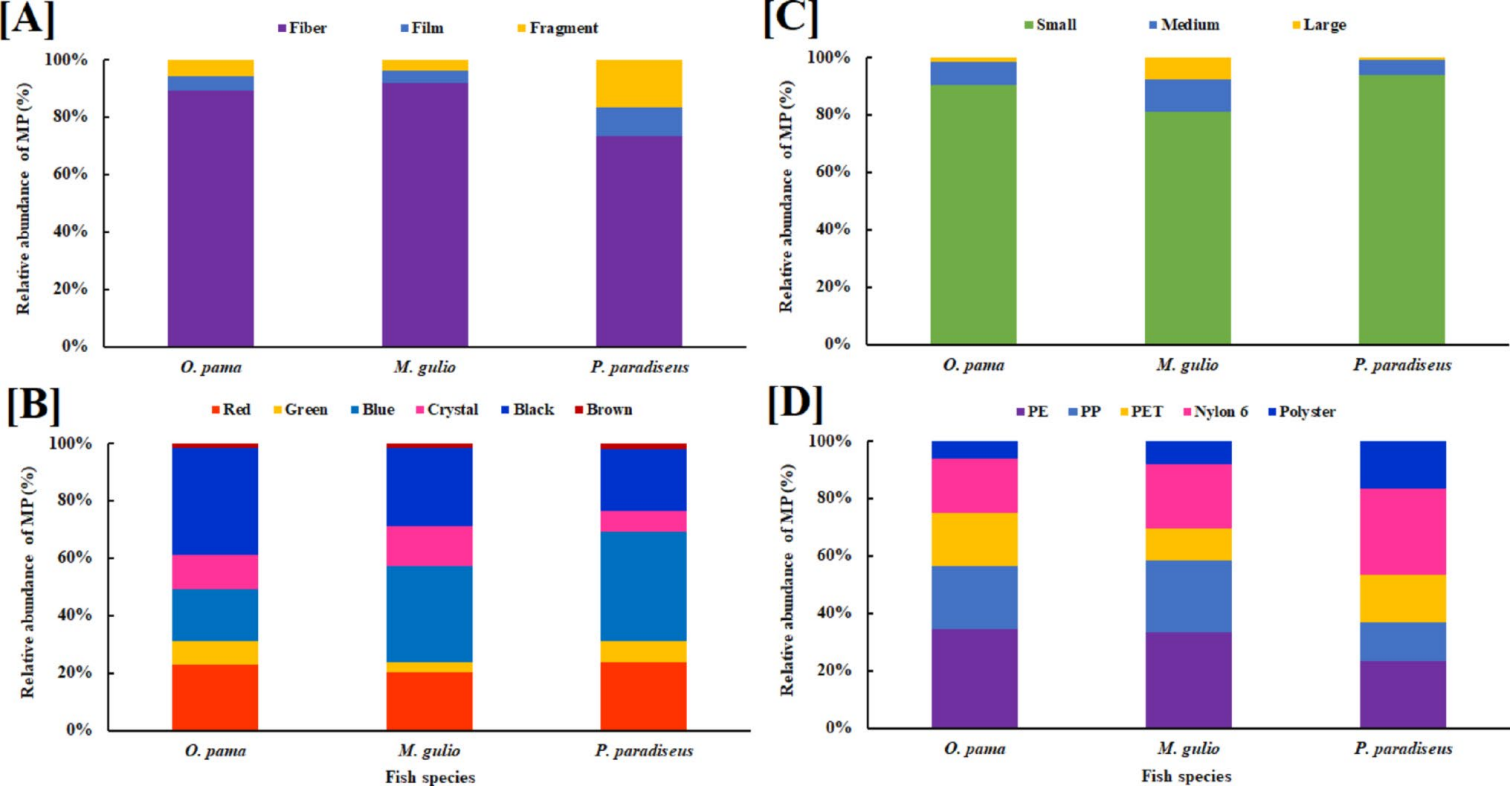
Relative abundance of MPs in *O. pama*,* M. gulio*, and *P. paradiseus* by **A** shape, **B** Colour, **C** size range (small = < 500 μm, medium = 500–1000 μm, and large = 1000–5000 μm), and **D** polymer types (PP = polypropylene, PE = polyethylene, PET = polyethylene terephthalate, PS = polystyrene or polyster, and Nylon 6)

Three different polymer fiber, film, and fragment forms were discovered (Fig. 3), where fibers were predominant in all fishes and ranged from 73.20 to 91.75% of the total MPs (Fig. 4A). Fishes ingested a wide range of colors, predominantly black, green, blue, crystal, red, and brown. Blue, red, and black were the most common colors, representing 18.38–38.14%, 20.39–23.71%, and 21.65–37.50% of the total MPs (Fig. 4B). MPs were sorted into three size classes. Small-size MPs (500–1000 μm) were most frequent in the fish GITs, accounted 81.07–93.81%, followed by medium (5.15–11.17%) and large-size MPs (1.03–7.77%) (Fig. 4C). The present study identified five distinct polymers, polyethylene (PE, 23.33–34.38%), polypropylene (PP, 13.33–25.0%), polyethylene terephthalate (PET, 11.11–18.75%), Nylon 6 (18.75–30.0%), and polystyrene or polyster (PS, 6.25–16.67%) from the extracted samples (Fig. 4D). FTIR is one of the reliable techniques to confirm the existence of MPs. Thus, FTIR analysis was used to identify the polymers in a subset of the common MP types (polypropylene (PP), polyethylene (PE), polyethylene terephthalate (PET), polystyrene (PS) and Nylon 6. The percentage of polymer in these fish samples were PE (23.33–34.38%), PP (13.33–25.0%), PET (11.10–18.75%), Nylon 6 (18.76–30.05) and PS (6.25–16.67%) (Fig. 4D). FTIR spectra of the identified polymers and their reference spectra are presented in supplementary Fig. S1.

### Relationship of MP Abundance with Total Length, Body Weight, and GIT Weights of Fishes

**Fig. 5:**
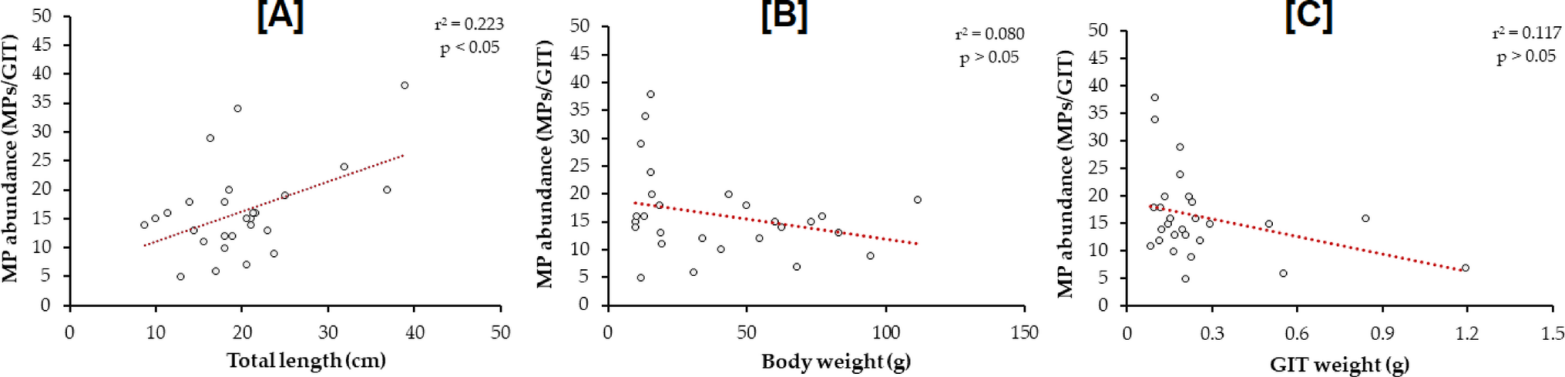
The relationships among MP abundance (MPs/GIT), total length (cm), body weight (g), and GIT weight (g) of fish samples

The MP incidence was significantly correlated with the total length of fish (r^2^ = 0.223, *p* = 0.013) (Fig. 5A). In contrast, no significant relationships were identified between MP abundance and body weight (r^2^ = 0.080, *p* = 0.153), and MP abundance and GIT weight (r^2^ = 0.117, *p* = 0.080) of fish samples (Fig. 5B and C).

### Polymer Hazard Index

The PHI of fish species ranged between 512 and 701, which fall under the risk category of “Danger” (100 < PHI < 1000 = hazard category IV) (Table 2). The hazard scores of Nylon 6 and PS were 50 and 30, respectively. Therefore, increasing the abundance of high-hazard polymers, such as Nylon 6 and PS, increases the PHI scores.

**Table 2:** Polymer hazard index of three estuarine fish species from the Meghna river estuary

Fish species	Polyethylene (PE)	Polypropylene (PP)	Polyethylene terephthalate (PET)	Nylon 6	Polystyrene (PS)	PHI	Hazard level	Hazard Category
*Otolithoides pama*	121	7	24	300	60	512	High hazard	IV
*Mystus gulio*	132	9	16	400	90	647	High hazard	IV
*Polynemus paradiseus*	77	4	20	450	150	701	High hazard	IV

## Discussion

MP pollution represents a critical threat to aquatic ecosystems and has garnered substantial attention due to its pervasive presence in global water bodies. MP intake by fish poses a notable concern as it facilitates the biomagnification of MPs through the food chain, ultimately affecting human health. Reflecting this urgent environmental issue, the current study delves into the abundance and distribution of microplastics by examining the gastrointestinal contents of three economically important brackish water fish species from the Meghna estuary of Bangladesh.

While discussing other studies around the world suggest that the average MP load per individual in this study is higher from the fish of the Northwest Peninsular, Malaysia (Foo et al. 2022), the northwest coast of India (Prusty et al. 2023), the west coast of Kyushu, Japan (Yagi et al. 2022), the western coast of the Black Sea, Turkey (Atamanalp et al. 2021), the Mondego estuary, Portugal (Bessa et al. 2018), the Goiana Estuary, South America (Ferreira et al. 2018), the island of Newfoundland, Canada (Liboiron et al. 2019), the coast of Louisiana, USA (Gad and Midway 2022), the Pra estuary, Ghana (Amponsah et al. 2024) and from Fiji and Australia (Wootton et al. 2021). In contrast, the research conducted by Siddique et al. (2022) on the Hilsa shad from the lower Meghna River estuary showed a higher abundance of MPs than the present study findings. However, fiber was the most predominant shape of MPs found in the fish GIT, and polyethylene polymer was the most common chemical composition in most cases (Table S1).

MPs were consistently detected across all studied fishes, with fibers emerging as the predominant MP type. This finding resonates with broader environmental research indicating that fibers are the most prevalent MP form in aquatic ecosystems, with its fauna, mainly fish, being significantly impacted (Hossain et al. 2019; Siddique et al. 2022). These fibers often originate from several anthropogenic sources, including the degradation of fishing gear, effluent discharge from the textile industry, and residues from domestic laundry, underscoring the significant environmental footprint of human activities (Browne et al. 2011). The Meghna River estuary is rich in fisheries resources and plays a vital role in supplying a large quantity of fish to both local and national markets, as well as providing livelihoods for local communities (Siddique et al. 2021). Artisanal fishing in this estuary contributes significantly to MP pollution through intensive fishing practices. Discarded fishing nets, a major source of marine debris, pose a severe threat to the marine environment and contribute to plastic pollution due to the synthetic polymers they contain (Chowdhury et al. 2024). Erosion is a common occurrence in the lower Meghna River estuary (Das et al. 2020), and the continuous erosion in this estuary results in large amounts of land-based plastics and MPs entering the water, further exacerbating MP pollution. Fibrous MPs are particularly concerning due to their heightened carcinotoxic potential and prolonged retention in the organism’s digestive tract compared to other MP types (Lei et al. 2018). Fiber-type MPs are especially concerning due to their physical properties and persistence in the environment. Once ingested by fish, these fibers can accumulate and cause physical and chemical impacts, potentially affecting the health and survival of these organisms (Rochman et al. 2013). Furthermore, ingesting microplastic pieces by fish species is not merely an environmental health issue but also raises concerns about food safety for humans consuming fish and fish products. Studies have demonstrated that microplastics can transfer from the GIT to the muscle tissue of fish, though the extent of this translocation and its implications for human consumption are still under investigation (Carbery et al. 2018).

The color of MPs revealed a diverse palette, with blue, red, and black being predominant. This diversity suggests that fish do not selectively ingest MPs based on color. Fish usually ingest colorful MPs during light conditions, and the ingestion of colorful MPs declines during dark conditions (Okamoto et al. 2022). MPs color findings of the present study are parallel to those of Prusty et al. (2023), who identified black and blue as the most prevalent color of MPs in *H. nehereus* GIT from the northwest coast of India, and *P. lethostigma* from the Louisiana coast also found a majority of blue and black MPs (Gad and Midway 2022). The diversity in colors and types of MPs found in fish GITs, as observed in various studies (Table S1), including the present study, indicates a broad spectrum of sources and pathways through which these pollutants enter aquatic ecosystems. Moreover, fishes’ predilection for ingesting MPs resembling their natural prey in color underscores the deceptive danger posed by MPs, echoing their potential to mimic natural food sources and thereby increase ingestion rates (Roch et al. 2020).

The polymers detected, namely PE, PP, PS, nylon 6, and PET, are common in various fishing apparatus, highlighting a direct link between fishing activities and MP pollution (Siddique et al. 2022). The absorption characteristics of these polymers, particularly for toxic compounds like chlorinated benzenes and PAHs, raise significant concerns regarding their role in contaminant bioaccumulation within aquatic food webs (Kibria et al. 2023). According to Wootton et al. (2021), PE was the most common polymer found in their studied fishes, the same as the most abundant polymer type in this study. People can intake compounds from absorbed plastic and plastic additives by consuming MP-contaminated seafood. Identifying these polymer types can be used to assess the additives present in the MPs and can play a significant role in future research.

Our study found no significant correlation between the number of MPs and the fish’s body weight or GIT weight, aligning with observations by Roch et al. (2020). However, a clear relationship was established between fish length and MP load, supporting similar findings in Khan and Setu (2022) research on the Jamuna River fish in Bangladesh. Mercy et al. (2023) found a significant relationship between the microplastic intake of fish and their body weight and length in major urban lakes of Dhaka, Bangladesh. They suggested that the abundance of microplastics in fish is more closely related to body size than to the intensity of plastic pollution in the surrounding environment. Mercy and Alam (2024) also observed a significant correlation between the abundance of microplastics in shrimps and their body weight or length in the Bay of Bengal. Another study indicated a strong correlation between length, depth, and weight, with the size of microplastics being negatively correlated with these growth parameters (Rahman et al. 2024). This suggests that the impact on growth (length, depth, and weight) diminishes as the size of the microplastics decreases (Rahman et al. 2024).

Microplastics are considered a threat to aquatic environments due to their various harmful effects on human health, the environment, and economic stability (Karami et al. 2017). Microplastics in seafood seriously threaten food safety; however, it is yet unclear what concerns these MPs may represent to human health (van Cauwenberghe and Janssen 2014). PHI is a crucial criterion when evaluating the risk of MPs (Nithin et al. 2022). According to Nithin et al. (2021), the PHI of microplastic contamination in various fishes taken from the Southeast coast of India was 6.27, 3.4, and 2.7 for LDPE, PP, and PS, respectively. These fishes were classified in the hazard category II (medium risk) based on their PHI scores ranging from 1 to 10. Because of the high levels of hazardous polymers like PS and Nylone 6, the PHI of the fish species we chose for this investigation is significantly greater than that of the Nithin et al. (2022) study.

The potential of plastics to host diverse microbial communities through biofilm formation presents an additional vector for pathogen transmission, further complicating the health implications of MP pollution (Siddique et al. 2022). This aspect underlines the necessity for a more integrated understanding of MP pollution, considering its ecological impacts and potential to adversely influence human health. By augmenting the discourse on MP pollution with these detailed insights, the study underscores the multifaceted risks posed by MPs, advocating for informed strategies to mitigate this pervasive environmental challenge.

## Conclusion

MP pollution has increasingly become a grave concern worldwide. This research evaluated MPs concentration in the gastrointestinal tract of three brackish water fish species to discern the association between MPs and the fish’s body length and weight. The study unveiled a positive relationship between the quantity of MPs and the fish’s weight and length. Although MPs were predominantly found in the gastrointestinal tract, a generally non-edible part of the fish, it is crucial to acknowledge that MP concentration escalates with the fish’s length and weight. Consequently, additional research is imperative to comprehend the magnitude of this issue comprehensively and to devise effective strategies for mitigating the potential adverse impacts on both aquatic life and human health.

## Supplementary Information

Below is the link to the electronic supplementary material.

Supplementary file (DOCX 120 KB)
