# Supplementary material for: Unveiling Microplastics in Commercial Brackish Water Fishes from the Lower Meghna River Estuary of Bangladesh
Source: Bull Environ Contam Toxicol. 2025 Apr 26;114(5):68. doi: 10.1007/s00128-025-04048-3 (PMC12031850; doi:10.1007/s00128-025-04048-3)
Supplement: Supplementary file 1 — Supplementary file (DOCX 120 KB) [file 128_2025_4048_MOESM1_ESM.docx]

**Supplementary Information**

| 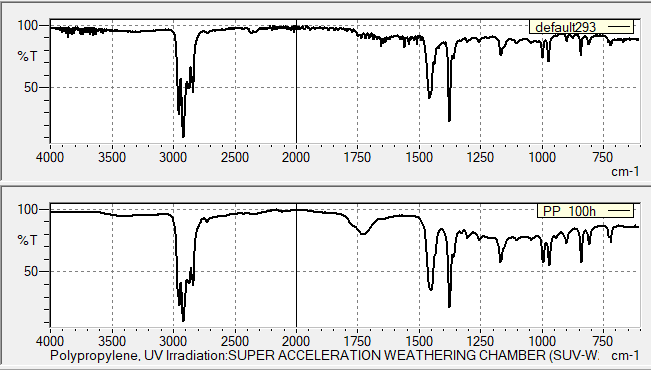  (A) Polypropylene (PP) | 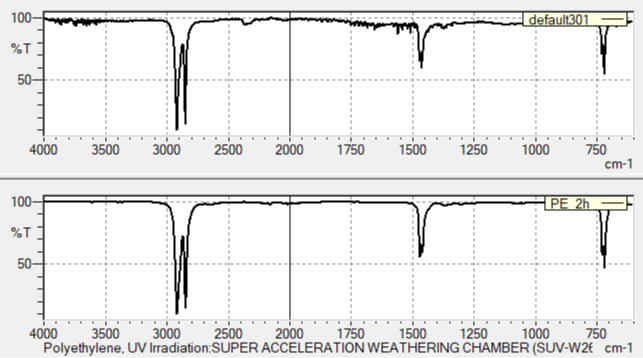  (B) Polyethylene (PE) |
| --- | --- |
| 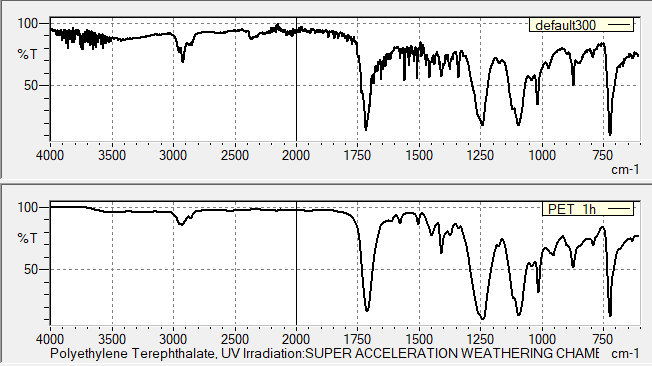  (C) Polyethylene Terephthalate (PET) | 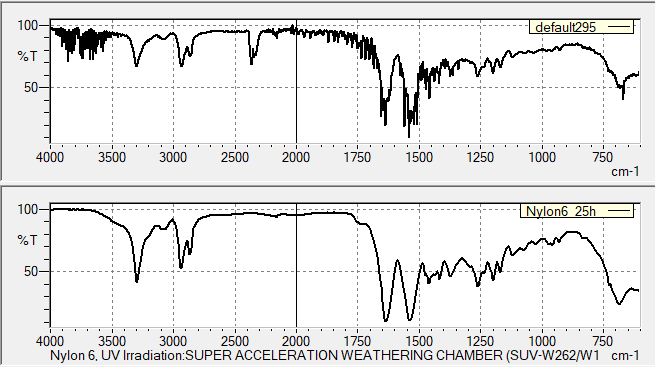  (D) Nylon 6 |
| 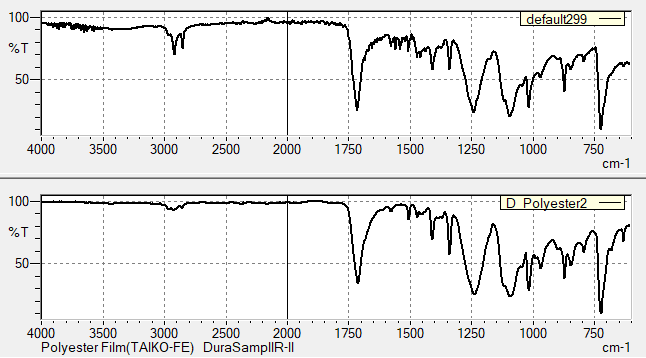  (E) Polystyrene (PS) | |

**Fig. S1**: FTIR spectra of identified polymers of MPs and their reference spectra (presented on the bottom).

**Supplementary Table S1**: Microplastics found in fish GIT around the globe. PET- Polyethylene terephthalate, PE- Polyethylene, PS- Polystyrene, PU- Polyurethane, PVC- Polyvinyl chloride, PP- Polypropylene, PAN- Polyacrylonitrile

| **Country** | **Scientific Name** | **Sample** | **Number of MPs** | **Dominant shape** | **Dominant colours** | **Dominant polymer** | **Reference** |
| --- | --- | --- | --- | --- | --- | --- | --- |
| Present study |  |  |  |  |  |  |  |
| Bangladesh | *Tenualosa Ilisha* | 15 | 287 | Fiber | Transparent | - | Siddique et al., 2022 |
| Malaysia | *Atule mate*  *Crenimugil seheli*  *Sardinella fimbriata*  *Rastrelliger brachysoma* | 72 | 432 | Fragment | - | PET | Foo et al., 2022 |
| India | *Harpadon nehereus* | 213 | 3409 | Thread | Black, Blue | PE, PS, PU | Prusty et al., 2023 |
| Japan | *Scomber japonicus*  *Trachurus japonicus*  *Zeus faber*  *Macroramphosus scolopax*  *Carangoides equula* | 321 | 501 | Fiber | Transparent, Blue | PE, Fiber | Yagi et al., 2022 |
| Turkey | *Mullus barbatus* | 82 | 62 | Fiber | Black | Poly-chloroprene | Atamanalp et al., 2021 |
|  | *Alosa immaculata* | 82 | 103 |  |  |  |  |
| Portugal | *Dicentrarchus labrax*  *Diplodus vulgaris*  *Platichthys flesus* | 120 | 157 | Fiber | Blue | Polyester | Bessa et al., 2018 |
| Brazil | *Cynoscion acoupa* | 552 | 1073 | Filaments | Blue | - | Ferreira et al., 2018 |
| Canada | *Gadus morhua* | 1010 | 19 | Fiber | White | PE | Liboiron et al., 2019 |
| USA | *Ariopsis felis* | 40 | 8 | Fragment | Blue | PP | Gad & Midway, 2022 |
|  | *Paralichthys lethostigma* | 50 | 16 | Fiber | Blue, Black | PS, PAN |  |
| Australia | *Plectropomus leopardus*  *Upeneichthys lineatus*  *Lutjanus gibbus*  *Mugil cephalus* | 73 | 110 | Fiber | - | PE | Wootton et al., 2021 |
| Fiji | *Plectropomus leopardus*  *Lutjanus gibbus*  *Parupeneus indicus*  *Mugil cephalus* | 120 | 102 | Film | - | PE |  |
